# Suitability of European *Trichogramma* Species as Biocontrol Agents against the Tomato Leaf Miner *Tuta absoluta*

**DOI:** 10.3390/insects11060357

**Published:** 2020-06-08

**Authors:** Lea Schäfer, Annette Herz

**Affiliations:** Institute for Biological Control, Julius Kühn-Institute, Heinrichstr. 243, D-64287 Darmstadt, Germany; schaefer-lea94@gmx.de

**Keywords:** biological control, host acceptance, host preference, invasive pests, species identification, tomato crops, *Trichogramma* species, *Tuta absoluta*

## Abstract

The tomato leaf miner *Tuta absoluta*, originally from South America, is an invasive species threatening European tomato crops. Since various insecticides have become ineffective in controlling *T. absoluta*, effective and environmentally friendly alternatives are needed. Biological control, especially by *Trichogramma* parasitoids, is considered to be an effective means of reducing this pest. Thus, the aim of our study was to identify promising candidates of *Trichogramma* parasitoids for biological control of *T. absoluta* in Europe. We assessed the efficiency of nine European *Trichogramma* species and compared them to *Trichogramma achaeae*, as this species is already commercially available. Firstly, we verified species identity of the 10 rearing strains using molecular and morphological methods. Then, host acceptance, host preference (*T. absoluta* vs. rearing host *Sitotroga cerealella* eggs) and host searching capacity were tested under laboratory conditions. Our results indicated that *T. nerudai*, *T. pintoi* and *T. cacoeciae* achieved a similar level of parasitism on potted tomato plants as *T. achaeae*. For the next step, these promising strains should be tested under greenhouse conditions.

## 1. Introduction

Increasing globalization and environmental changes facilitate the spread and establishment of invasive species [1,2]. Due to the absence of natural enemies, growth of invasive populations is promoted, resulting in major losses in agriculture [1]. The tomato leaf miner *Tuta absoluta* Meyrick (Lepidoptera, Gelechiidae) is native to South America and was introduced to Europe in 2006 [3]. In Germany, *T. absoluta* was first detected in 2009 in several tomato production sites [4]. Larval feeding damages all above-ground parts of the plant, reduces photosynthetic capacity, and consequently growth and yields [3,5,6]. In addition, infested plants are more vulnerable to secondary diseases caused by fungi and bacteria [5]. If no pest control strategies are taken, *T. absoluta* infestation can cause a total crop loss [6,7]. Currently, control of *T. absoluta* is mainly based on chemical treatments [8,9]. Besides major risks for the environment, non-target organisms [10,11,12,13], and human health [14,15], the intensive use of pesticides promotes the development of resistances. Due to their mine-feeding behavior, *T. absoluta* larvae are less susceptible to chemical control and frequent applications are necessary [16]. As a result, resistances against various active ingredients have been recorded [9,12,17,18,19]. For instance, plant protection products based on chlorantraniliprole and spinosad that are authorized in Germany have become ineffective in controlling the pest [20]. Moreover, European Community (EC) legislation has limited the availability of active ingredients. This will further reduce the use of conventional pesticides in the European Union [21]. Hence, alternative strategies are required that are both effective and environmentally friendly.

Biological control is a key component of integrated pest management (IPM) and allows a selective and sustainable crop protection. *Trichogramma* (Hymenoptera, Trichogrammatidae) egg parasitoids are currently utilized successfully in biocontrol programs against several lepidopterous pests [22,23,24,25]. Adult female parasitoids attack the egg stage of their host and kill the target pest before crop damage by larval feeding commences [26]. In some Southern European and North African countries, *Trichogramma achaeae* Nagaraja & Nagarkatti is currently used for inundative biological control of *T. absoluta* [27]. However, the production of *T. achaeae* is difficult and expensive due to the absence of thelytoky and diapause [28]. In addition, *T. achaeae* is not a native species in Europe, even if its occurrence has been reported in the Canary Islands and Azores. Nonetheless, *T. achaeae* was introduced to mainland Spain and France for use in biological control of *T. absoluta* [29]. Commercial production and use of this non-native species is not practiced in many countries throughout the EU, including Germany, where the current legislation based on the Federal Nature Conservation Act sets some constraints to field releases of non-native organisms [30]. Since *T. achaeae* seems to be a good colonizer and is highly adaptive, an invasion into native ecosystems is possible [6,31]. To date, no European *Trichogramma* strains have been identified as suitable alternatives to *T. achaeae* [26,28].

Thus, the aim of the present study was to identify candidates for biological control of *T. absoluta* in Europe. We investigated the efficiency of 10 *Trichogramma* rearing strains, including four species native to Germany. Some of the selected species also occur in the original distribution or invasion area of the tomato leaf miner or have already been found in tomato crops. Accordingly, we expected a high affinity to the target system *T. absoluta*/tomato plant. *Trichogramma achaeae* was used as a control species as it is already commercially available.

This study is divided into two main parts: In the first part, species identity of the rearing strains was verified using molecular and morphological methods. In the second part, host acceptance of the target host *T. absoluta* and host preference (vs. the rearing host *Sitotroga cerealella*) were tested in a laboratory screening. Parameters assessed included parasitoid performance (level of parasitism, proportion of females parasitizing the target eggs), host-feeding, and host suitability (emergence rate, sex ratio). Based on these findings, we selected four promising species and investigated host searching capacity on potted tomato plants.

## 2. Materials and Methods

### 2.1. Biological Material

Tomato plants (*Solanum lycopersicum*, variety Alissa F1, Ruby Red) were grown in a greenhouse (25 ± 1 °C, night temperature 15 ± 1 °C, 16:8 h light:darkness (L:D)) and were watered every second day. Seed trays with drainage openings (60 × 40 × 6 cm) containing Bio-Potgrond (Klasmann-Deilmann GmbH) as substrate were used for sowing. Seedlings (2–4 weeks old) were placed individually in plastic pots (9 × 9 × 8 cm, Soparco) filled with Fruhstorfer soil type LD 80 (HAWITA Gruppe GmbH).

The tomato leaf miner *Tuta absoluta* was reared in nylon cages (60 × 60 × 60 cm, mesh size: 150 × 150 µm, BugDorm Megaview Science Co., Ltd., Taichung, Taiwan), on potted tomato plants, inside a rearing room (c.a. 23 ± 2 °C, 16:8 h L:D). Adult moths were supplied with honey, sugar solution (15% sucrose), and water. Honey was offered because it consists of various sugar types and pollen. Since honey dries out rapidly and then becomes too viscous for adult moths, sugar solution was offered as a basic supply. Sugar solution and water were offered in sealed plastic cups (500 mL), provided with a sponge cloth. Honey was spread thinly on a piece of Parafilm^®^, attached to one wall of the cage.

Egg parasitoids of the genus *Trichogramma* were reared on *Sitotroga cerealella* (Olivier 1879) eggs (BIOCARE GmbH, Dassel-Markoldendorf, Germany) at 25 ± 1 °C and at a photoperiod of 16:8 h L:D. In Germany, *S. cerealella* is used as factitious host for commercial production of *Trichogramma*. Rearing was carried out in glass tubes (length: 14.5 cm, diameter: 2.8 cm), sealed with a cotton fabric, and parasitoids were fed honey agar (3 g agar–agar, 100 mL dH_2_O, 200 g honey [32]).

### 2.2. Molecular and Morphological Identification

In this study we used 10 *Trichogramma* strains (Table 1) that are part of the stock collection of the Institute for Biological Control, Darmstadt, Germany. Since laboratory cultures of *Trichogramma* are prone to contamination by other species [33], we verified species identity through molecular and morphological methods. Molecular identification was carried out following the approach described by Silva et al. (1999) [34] and Stouthamer et al. (1999) [35]. Species were identified according to the size and the sequence of their ITS2 (internal transcribed spacer 2) PCR product of rDNA. For each strain, five females (frozen at −18 °C) were ground in distilled water. After addition of Proteinase K (20 mg/mL, biotechrabbit), samples were incubated overnight at 56 °C, followed by 10 min at 95 °C. PCR amplification was conducted using the QUIAGEN (Hilden, Germany) *Taq* PCR Core Kit and the primers 5′-TGT GAA CTG CAG GAC ACA TG-3′ (forward) and 5′-GTC TTG CCT GCT CTG AG-3′ (reverse) [35]. PCR products were electrophoresed on a 2% agarose gel (MetaPhorTM agarose, Biozym Scientific GmbH, Hessisch Oldendorf, Germany) and visualized under UV light (ChemoCam, Intas Science Imaging Instruments GmbH, Göttingen, Germany) by means of DNA stain MidoriGreen (Biozym Scientific GmbH). Molecular weight standards (Gel Pilot 100bp Plus Ladder, QUIAGEN) were used as reference. PCR products were purified using the QUIAGEN MinElute PCR Purification Kit, following the manufacturer´s instructions, and sequenced in both directions by StarSEQ GmbH (Mainz, Germany). Sequences were aligned manually using Geneious Prime 2019.1.2 and compared to the ITS2 sequences of known *Trichogramma* spp. published in GenBank by BLAST analysis.

Morphological identification was made based on differences in male genital structures [36,37]. Males (frozen at −18 °C) from arrhenotokous rearing strains were incubated in lactic acid (90%) for one week at room temperature. Then, whole body mounts were prepared in Hoyer´s medium. Dried specimens were examined under a Zeiss Axioskop light microscope (400× magnification) and photographed using the microscope software ZEN lite (2012). Most species were characterized using an identification key of the European *Trichogramma* fauna [38]. Morphological terminology follows Pinto (1998) [39].

### 2.3. Host Acceptance and Host Preference

No-choice tests were carried out in order to investigate whether the *Trichogramma* strains accept and parasitize the eggs of the target pest on tomato foliage. Ten *T. absoluta* eggs (<24 h) were placed on each of 10 punched out tomato leaf discs (variety Alissa F1, Ø = 1.2 cm). Prepared leaf discs were then transferred into small transparent cube-shaped plastic vessels (2 × 2 × 2 cm). Then, single females of a particular strain (<24 h, naïve (no oviposition experience), mated, fed honey agar, n = 30 per strain) were added to the leaf disc-equipped plastic cubes. *Trichogramma* females used in the tests have been separated from males based on their antenna morphology before [41]. Egg contacts were determined by observing parasitoid females for two hours at 5-minute-time intervals using a stereomicroscope (24 observations/female in total). Subsequently, parasitoid females were removed, and leaf discs were transferred individually into medication cups for further incubation at 25 °C, 70% RH, 16:8 h L:D. After seven days, we counted the number of parasitized, black (melanized) eggs, the number of unhatched yellow-colored eggs, and the number of *T. absoluta* larvae. We also recorded the proportion of parasitoid females that parasitized at least one *T. absoluta* egg (defined as active females), as suggested by [28]. After *Trichogramma* emergence, the number of adults and the sex ratio of F1 generation were determined.

Choice tests were carried out to investigate whether *Trichogramma* strains prefer the eggs of the target or rearing host. Five *T. absoluta* eggs (<24 h) and five *S. cerealella* eggs (cold stored at 7 °C) were glued in alternating order (distance = 5 mm) with the botanical glue tragant (Merck, Darmstadt, Germany, prepared as 3% solution in tap water) onto a piece of paper (2 × 2 cm). These paper sheets with eggs were exposed to single *Trichogramma* females (< 24 h, naïve, mated, fed honey agar, n = 30 per strain) in plastic cubes for two hours. Egg contacts were recorded in the same way as during acceptance tests described above. Eggs of *T. absoluta* and *S. cerealella* were incubated separately in medication cups at 25 °C, 70% RH, 16:8 h L:D. We recorded the number of parasitized eggs, aborted, undeveloped eggs, and hatched host larvae. The proportion of females that parasitized at least one *T. absoluta* or one *S. cerealella* egg was also calculated. In addition, we determined the number of emerged *Trichogramma* adults and sex ratio of F1.

Both acceptance and preference tests were conducted in the laboratory at 25 °C in a humidity tray. For this, a wet paper towel was placed at the bottom of a plastic box and covered with a wire mesh, which carried a smaller plastic tray containing the ten cubes. Almost homogeneous illumination was achieved using a fiber optic illuminator (SCHOTT), as suggested by [42]. In both laboratory screening tests (host acceptance and host preference) 10 females of each strain were tested in parallel. Since each trial was carried out three times, 30 females per strain were tested in total. We also checked egg survival in the absence of parasitoids (control condition) to assess natural egg mortality. Length and width of twenty eggs of each host species were measured using a Zeiss Axio Zoom V16 microscope in order to detect morphometric differences between *T. absoluta* and *S. cerealella* eggs.

### 2.4. Host Searching Capacity

Based on our findings of the laboratory screening, we selected four promising *Trichogramma* strains (ACA, NER, PIN, CAC) for further tests on potted tomato plants. Thirty *T. absoluta* eggs (<24 h) were glued with tragant individually on each of four potted tomato plants, variety Ruby Red. Half were distributed on upper and half on lower leaf faces. Prepared tomato plants were placed individually in acrylic glass cylinders (height = 40 cm, diameter = 20 cm) that were sealed with a cotton fabric to allow air circulation. Honey agar was used as food source for parasitoids. Twenty *Trichogramma* females (<24 h, naïve, mated, fed honey agar) per strain were released from centrifuge tubes (height = 7.5 cm, diameter = 1 cm) in a defined parasitoid: host ratio (1:1.5) at the base of the tomato plant. Experimental cylinders were randomly assigned to the four species by lot. Trials were carried out at 23 °C, 70% RH, 16:8 h L:D and were performed six times to obtain six replicates for each strain. After 48 h, *T. absoluta* eggs were cut out together with some leaf material, transferred into medication cups and incubated at 25 °C, 70% RH, 16:8 h L:D. Seven days after the start of the experiment, we recorded the number of parasitized eggs, aborted eggs, and hatched *T. absoluta* larvae. Searching capacity on the host plant was determined indirectly via the level of parasitism.

### 2.5. Statistical Analysis

All statistical analyses were performed using RStudio software (version 1.1.453, RStudio Team 2016). Data were checked for homogeneity of variance (F test, Levene test) and normal distribution of residuals (Shapiro–Wilk test). If parametric assumptions were not met, we used nonparametric tests. In the acceptance test, each dependent variable (“egg contacts”, “number of parasitized eggs”, “egg mortality”) was analyzed individually using Kruskall–Wallis tests with “*Trichogramma* strain” as independent variable. Then, a Dunn’s post hoc test with Bonferroni adjustment was conducted where omnibus tests were significant. The parameters “proportion of active females”, “emergence rate” and “sex ratio” were evaluated descriptively according to the IOBC (International Organisation for Biological Control) guidelines [43]. Host preference was analyzed using a Wilcoxon signed-rank test. Here, parasitism rates were compared within one *Trichogramma* strain depending on host species (*T. absoluta* vs. *S. cerealella*). A *t*-test was used to determine whether the egg lengths and widths of the two hosts species differ. Finally, host searching capacity was analyzed using one-way ANOVA with the dependent variable “percent parasitism” and “*Trichogramma* strain” as independent variable.

## 3. Results

### 3.1. Molecular and Morphological Identification

Based on the size and sequence of amplified ITS2 PCR products, 10 different species were identified. The size of the amplified PCR products ranged between 500 and 900 bp. Species identity of eight rearing strains was verified according to BLAST analysis (Table 2). However, species identity of two strains (COR, PIC) was not confirmed with molecular methods (Table 2).

Species identity of arrhenotokous rearing strains was verified using morphological methods. Seven strains were characterized using an identification key of the European *Trichogramma* fauna. Due to morphological differences in male genital capsule, strains were assigned to certain groups and then determined at species level (Figures S1–S5). *Trichogramma achaeae* and *T. nerudai* were not included in the identification key used. Thus, species identity of ACA (Figure S5A) was confirmed using a previous research paper [31]. NER was assigned to the *perkinsi (=parkeri)* group based on morphological characteristics (Figure S5B).

### 3.2. Host Acceptance

**Figure 1:**
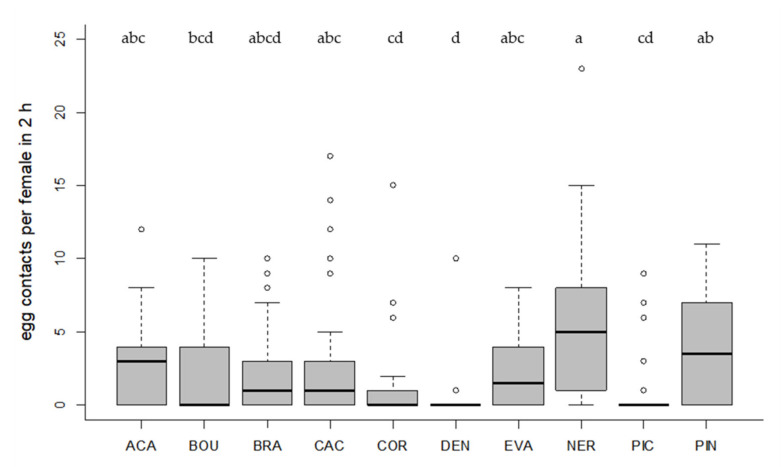
Number of contacts per parasitoid female with *T. absoluta* eggs in 2 h (24 observations/female in total). Box plots denote median (solid line), whiskers show range of data, and circles represent outliers. Different letters indicate significant differences between *Trichogramma* strains (*p* < 0.05, *n* = 30 females per strain).

**Figure 2:**
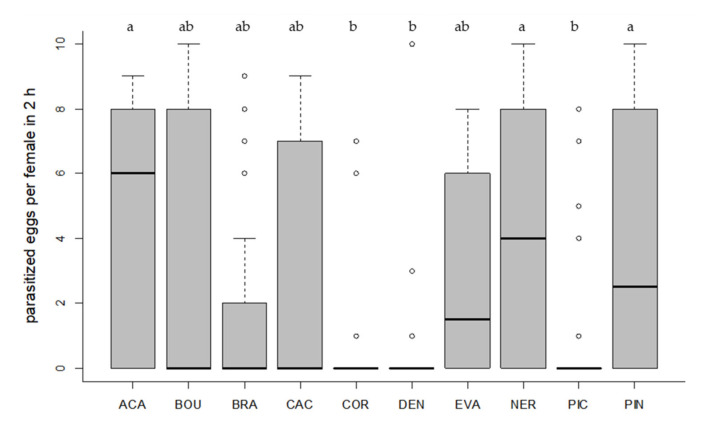
Number of parasitized *T. absoluta* eggs per parasitoid female. Ten eggs of the target host were offered to each female in a no-choice test on tomato leaf discs for two hours. Box plots denote median (solid line), whiskers show range of data, and circles represent outliers. Different letters indicate significant differences between *Trichogramma* strains (*p* < 0.05, *n* = 30 females per strain).

All tested *Trichogramma* strains accepted and successfully parasitized the eggs of the target host *T. absoluta* on tomato leaf discs (Figure 1 and Figure 2). Egg contacts varied significantly depending on *Trichogramma* strain (Kruskal–Wallis test, df = 9, X^2^ = 58.40, *p* < 0.001, Figure 1). Only one strain (DEN) showed significantly fewer egg contacts than the control species ACA (Dunn´s test, z = 4.24, *p* = 0.001, Figure 1). The level of parasitism also varied significantly depending on strain (Kruskal–Wallis test, df = 9, X^2^ = 53.85, *p* < 0.001, Figure 2). In general, strains with a high egg contact rate also had a high parasitism rate and vice versa (Figure 1 and Figure 2). The three strains (ACA, NER, PIN) with the highest level of parasitism differed significantly from the strains (COR, DEN, PIC) with the lowest parasitism rates (Dunn´s test, *p* < 0.05, Figure 2). Altogether, six strains did not differ significantly from ACA regarding their level of parasitism (*p* > 0.05, Figure 2).

We also determined the percentage of active females. In four strains (ACA, NER, PIN, EVA), more than 50% of the females parasitized at least one *T. absoluta* egg on tomato leaf discs in 2 h (Table 3). In three strains (COR, DEN, PIC), the proportion of females that parasitized was ≤20%. Mean emergence rates and sex ratios were used as indicators for host suitability. Emergence rates of parasitoids, developed in *T. absoluta* eggs, exceeded 80% in six strains (ACA, BOU, CAC, EVA, NER, PIN) (Table 3). The proportion of females in F1 was at least 50% in eight *Trichogramma* strains (Table 3). The offspring of the thelytokous strain CAC was exclusively female (Table 3). Two strains (EVA, DEN) produced more male offspring.

**Figure 3:**
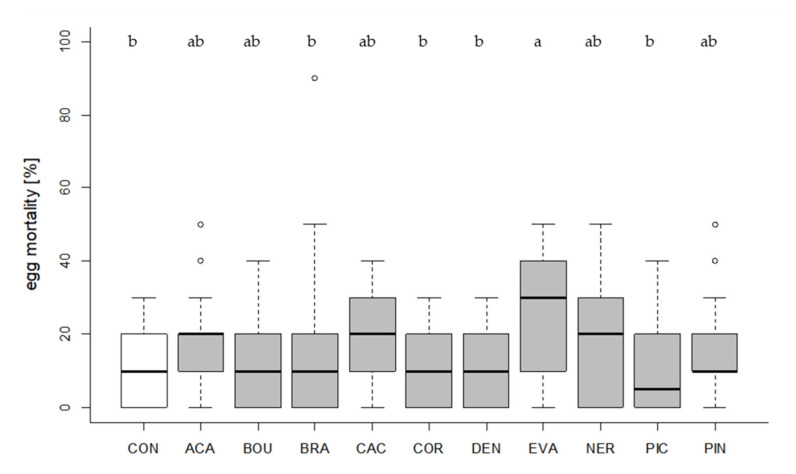
Percentage of aborted (=yellow colored, unhatched) *T. absoluta* eggs. Egg mortality of the control condition (CON, white, no *Trichogramma* exposure) and egg mortality depending on the tested *Trichogramma* strains (30 parasitizing females per strain provided with 10 eggs) are shown. Box plots denote median (solid line), whiskers show range of data, and circles represent outliers. Different letters indicate significant differences between groups (*p* < 0.05).

Some *Trichogramma* species may induce additional mortality of the target pest by host-feeding. Thus, we assessed whether the selected strains increased mortality of *T. absoluta* eggs compared to the control condition. Egg mortality differed significantly between groups (Kruskal–Wallis test, df = 10, X*^2^* = 42.056, *p* < 0.001, Figure 3). In one strain (EVA), egg mortality was significantly higher than in the control condition (Dunn´s test, z = 3.4799, *p* = 0.0276, Figure 3).

### 3.3. Host Preference

**Figure 4:**
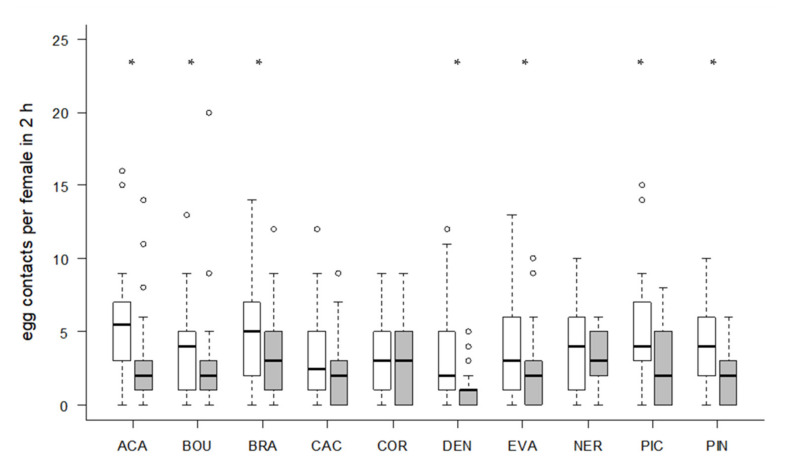
Number of contacts per parasitoid female with *S. cerealella* (white) and *T. absoluta* (grey) eggs in 2 h (24 observations/female in total). Box plots denote median (solid line), whiskers show range of data, and circles represent outliers. Asterisks indicate significant differences within one *Trichogramma* strain (*p* < 0.05, *n* = 30 females per strain).

**Figure 5:**
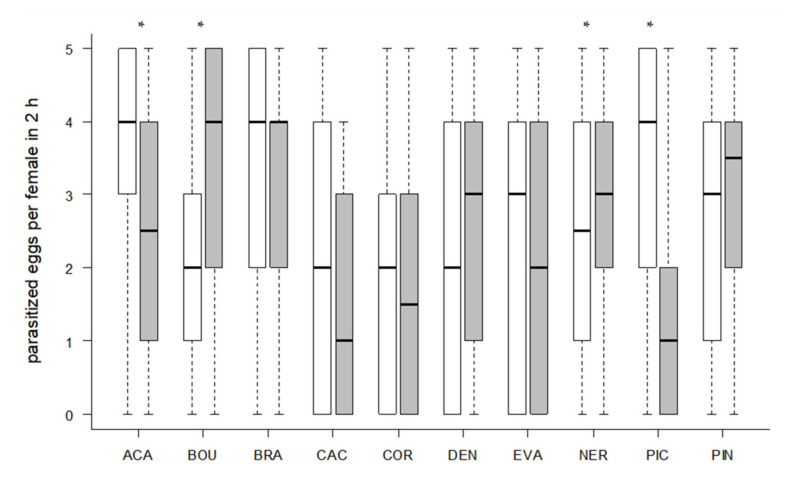
Number of parasitized eggs per parasitoid female. Five eggs of the rearing host *S. cerealella* (white) and five *T. absoluta* eggs (grey) were offered to each female simultaneously in a choice-test for two hours. Box plots denote median (solid line) and whiskers show range of data. Asterisks indicate significant differences within one *Trichogramma* strain (*p* < 0.05, *n* = 30 females per strain).

Significantly more contacts with the eggs of the rearing host *S. cerealella* were observed in seven strains (Wilcoxon signed-rank test, *p* < 0.05, Figure 4). However, only the two strains ACA (Wilcoxon signed-rank test, z = 2.5023, *p* = 0.0128) and PIC (z = 3.6498, *p* = 0.0019) parasitized significantly more *S. cerealella* eggs, whereas NER (z = 2.053, *p* = 0.0421) and BOU (z = 2.853, *p* = 0.0046) parasitized significantly more eggs of the target host *T. absoluta* (Figure 5). More than half of the females parasitized at least one *T. absoluta* egg in all tested strains (Table 4). The proportions of females that parasitized *T. absoluta* eggs were at least 80% in four strains (ACA, BRA, BOU, NER). More than 60% females were active on *S. cerealella* eggs in all strains (Table 4) and more than 80% of females parasitized at least one *S. cerealella* egg in four strains (ACA, BRA, PIN, PIC).

**Figure 6:**
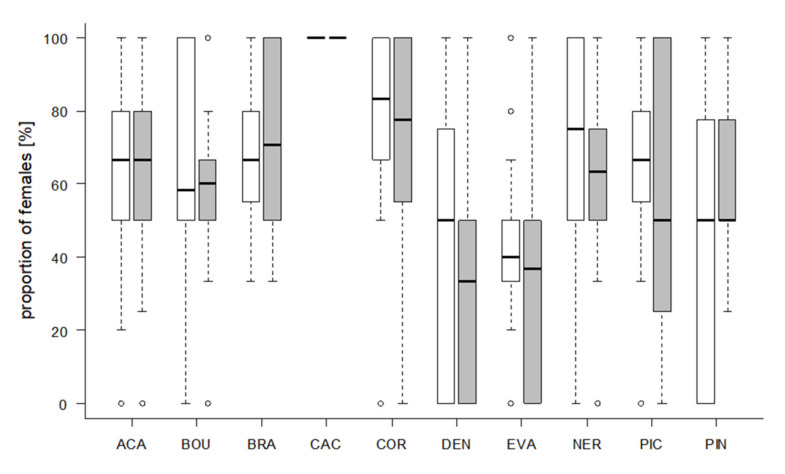
Percentage of female offspring per *Trichogramma* strain developed in *S. cerealella* (white) and *T. absoluta* (grey) eggs. Box plots denote median (solid line), whiskers show range of data, and circles represent outliers.

The proportion of female offspring was at least 50% in eight strains (Figure 6), independent of host species. Offspring of CAC was exclusively female. Two strains, DEN (host: *T. absoluta*) and EVA (both hosts) produced a higher number of males (Figure 6).

Egg sizes of the two different host species were compared by measuring egg lengths and widths. Eggs of *S. cerealella* were significantly longer than *T. absoluta* eggs (*t*-test, *t* = 30.967, *p* < 0.001, *n* = 20, Table 5). However, egg width did not differ between the two species (*t* = 1.2062, *p* = 0.2352).

Based on our findings of the laboratory screening, we chose two arrhenotokous strains for further tests on potted tomato plants in comparison to ACA: NER and PIN seemed to have a high affinity to the target system *T. absoluta*/tomato plant. Both strains achieved a similar level of parasitism than the control species ACA. In addition, both strains had a high emergence rate and female offspring was ≥50%. The strain NER even preferred the eggs of the target pest in a choice-test. In addition, we chose CAC because this strain is native to Germany, demonstrated a high emergence rate from *T. absoluta* eggs, and produced exclusively female offspring.

### 3.4. Host Searching Capacity

**Figure 7:**
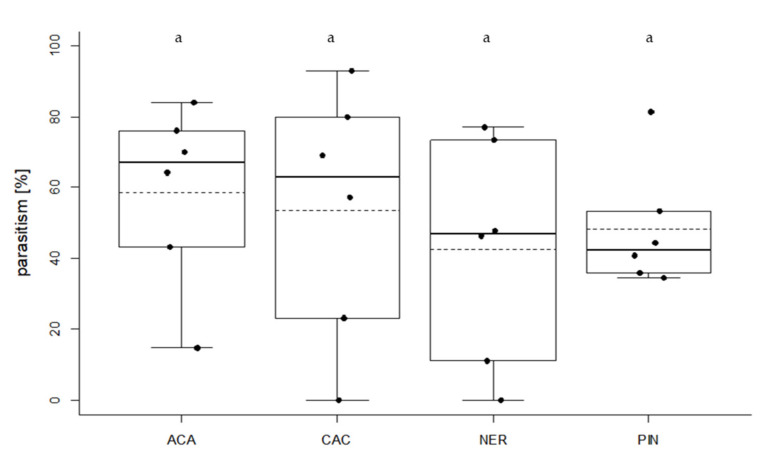
Host searching capacity of four *Trichogramma* strains on potted tomato plants (with 30 *T. absoluta* eggs/plant). Percentage of parasitized *T. absoluta* eggs at a parasitoid:host ratio of 1:1.5 is shown (six replicates). Whiskers denote range of data. In each box, medians are shown by horizontal solid lines and means by dotted lines.

All four tested strains were able to find and parasitize *T. absoluta* eggs on potted tomato plants (Figure 7). There were no significant differences in host searching capacity between the strains (ANOVA, df = 3, F = 0.3588, *p* = 0.7834, Figure 7). Mean level of parasitism was 58.7% for ACA, 53.7% for CAC, 42.6% for NER and 48.4% for PIN. Maximum level of parasitism was 84% for ACA, 92.9% for CAC, 76.9% for NER and 81.5% for PIN.

Overall, the design of the host acceptance test was suitable to predict the performance of the tested arrhenotokous strains on potted tomato plants. Nevertheless, the efficiency of the thelytokous strain CAC was underestimated based on the results of the laboratory screening.

## 4. Discussion

The results of the present study indicated that *T. nerudai*, *T. pintoi*, and *T. cacoeciae* can demonstrate a comparable performance in using eggs of *T. absoluta* as host and achieved a similar efficiency on potted tomato plants as the control species *T. achaeae*.

### 4.1. Molecular and Morphological Identification

Morphological identification is based on differences in male genital structure. Hence, species identity of the thelytokous strain CAC was only verified by molecular methods. In total, species identity of eight rearing strains was confirmed by BLAST analysis. Some of the ITS2 sequences in GenBank are only associated with generic names. Thus, species identity of COR was not confirmed with molecular methods. According to characteristics of male genital structure (e.g., posterior extension of dorsal lamina exceeds intervolsellar process, large apodemes [38,44]), the strain seemed to be *T. cordubensis*. However, morphological species identification is difficult without specialized skills [36], especially if no identification key is available. For that reason, the rearing strain NER could not be determined at species level using morphological methods, but was assigned to the *perkinsi* (=*parkeri*) group, consistent with [40]. Regarding *T. piceum*, no ITS2 sequences were available in GenBank. Even if BLAST analysis showed an almost 100% consensus with *T. lingulatum*, a species native to China and Japan [45], our rearing strain PIC could be clearly associated to *T. piceum* using the identification key of the European *Trichogramma* fauna. However, *T. piceum* and *T. lingulatum* share similar genital structures [46]. Although ITS2 is mostly useful in separating closely related species [35], exceptions are possible: for instance, *T. minutum* and *T. platneri* do not differ in their male genital structure [47] or their ITS2 sequence [48], but can be distinguished based on their COII sequence [49]. To ascertain whether *T. piceum* and *T. lingulatum* are distinct species, sexual compatibility should be tested with crossing experiments [50,51]. Release of the correct *Trichogramma* species is crucial for the success of biological control [35]. Moreover, an accidently introduced non-native species may have adverse effects on local fauna [52]. However, species identity of the four most promising strains (NER, PIN, CAC, and control species ACA) was clearly confirmed.

### 4.2. Host Acceptance and Host Preference

Host acceptance is considered the first critical step in the selection of suitable biocontrol agents. Two strains, NER and PIN, appeared promising regarding their level of parasitism. In general, parasitism rates significantly varied among species, as demonstrated in other screenings [26,28,42]. In addition, observed egg contacts were reflected in parasitism rates consistent with the results of previous studies [53,54]. However, a high egg contact rate does not necessarily result in a high parasitism rate, since thickness and structure of the chorion [55,56], as well as the host´s immune system [57,58], may prevent successful parasitism.

In general, the proportion of active females was relatively low in the host acceptance test. According to the recommendations in the IOBC guidelines, ≥80% of the females should parasitize at least one egg of the target pest in four hours [59]. One possible explanation for the obtained lower values is the short test duration of two hours, which was chosen due to the higher frequency of observation events and low number of eggs exposed to females. However, if the eggs were offered on a sheet of paper (host preference test), the number of active females was generally higher. Since parasitism levels are directly related to the proportion of females attacking the pest [26,28], both parameters are useful in measuring parasitoid performance. Low parasitism rates can be firstly explained by a rejection of the target eggs [28]. Most *Trichogramma* species tend to prefer large-sized eggs [60], while *T. absoluta* eggs are significantly shorter than the eggs of the rearing host *S. cerealella*. Secondly, some tested *Trichogramma* strains may not be able to cope with tomato plant trichomes [61,62,63,64,65,66]. Regarding *T. dendrolimi* and *T. cordubensis*, previous studies recorded a low affinity to the target system *T. absoluta*/tomato plant [28,67], consistent with our findings.

In six strains, emergence rate of parasitoids, developed in *T. absoluta* eggs, was at least 80%, indicating a high host-suitability. However, this trait is of minor importance since *Trichogramma* species are mostly released using the inundative method [23,45,68]. Tomato crops appear to be less favorable for the sustainable establishment of *Trichogramma* parasitoids [69]. The IOBC recommends an emergence rate of ≥80% to ensure efficient mass production on a rearing host [43].

A female-biased parasitoid sex ratio may benefit biological control, because only females parasitize and directly kill the target pest [45,70]. Thus, thelytokous species such as *T. cacoeciae* that produce exclusively female offspring may be favored [26]. In eight strains, the proportion of female offspring was at least 50%, as recommended by IOBC [43]. However, the two strains DEN and EVA produced a higher number of males. The sex ratio in hymenopteran parasitoids is influenced by multiple factors such as host species and host quality (e.g., size, age) [71,72,73,74]. In addition, the presence of PSR (paternal sex ratio) chromosomes or PSR-like elements results in a male-biased offspring [75,76,77]. Regarding *T. evanescens*, PSR-like elements have already been hypothesized [78].

Destructive host-feeding is common in hymenopteran parasitoids, including some Trichogrammatidae [79]. In one strain, EVA, the mortality of the host eggs was significantly increased compared to the egg mortality of the control, probably resulting from host-feeding. Destructive host-feeding is considered an additional effect in terms of biological control, particularly with inundative releases [80,81]. Thus, future studies should additionally determine larval reduction to evaluate parasitoid performance more precisely.

In general, host egg characteristics (e.g., size, quality, species) [82,83,84], as well as natal origin [85,86], may influence oviposition preference. In our study, preference measurement based on the two parameters (observed egg contacts, number of parasitized eggs) afforded different results. This can be explained by limitations of the first parameter in measuring preference: It is likely that parasitoid females needed more time to parasitize the larger eggs of the rearing host *S. cerealella*. A longer rest on a particular egg may result in an increased number of observed egg contacts for the respective host species. Previous studies have shown that the duration of parasitism behavior depends on host size [87] and species [88]. Because of its obvious limitation, the host contact parameter was not further considered for preference evaluation.

A preference for the target host *T. absoluta* is generally desired as it influences the parasitoid efficiency and reduces the risks on non-target organisms [83,89,90]. One of our most promising strains, NER, preferred *T. absoluta* eggs in a choice-test. However, this does not necessarily mean that *T. absoluta* eggs are preferred in the field [83,90]. Remarkably, the control species *T. achaeae*, which is already commercially available against *T. absoluta*, prefers the larger eggs of the rearing host.

### 4.3. Host Searching Capacity

Plant structural complexity [91,92], as well as leaf surface traits, e.g., trichomes [63,64,65,66], influence the foraging behavior of various *Trichogramma* species. Thus, we tested host searching capacity on potted tomato plants. *T. nerudai*, *T. pintoi*, and *T. cacoeciae* achieved a similar level of parasitism as the control species *T. achaeae*. Since no natural egg-laying took place, the presence of contact kairomones was presumably reduced. However, all strains were able to find and parasitize *T. absoluta* eggs on the host plant. Based on the results of the laboratory screening, we already expected a high affinity to the target system for *T. nerudai* and *T. pintoi*. Both *T. nerudai* [40] and *T. pintoi* [45] have been recorded in the Southern European invasion area of the tomato leaf miner and both species occur naturally in tomato crops [93,94]. Interestingly, *T. nerudai* also occurs in South America [39] and was found parasitizing *T. absoluta* eggs naturally in the field [95].

Regarding *T. cacoeciae*, the design of the laboratory screening was not suitable to predict the high level of parasitism on tomato plants. This can be explained by the short test duration (2 h) and the young age of the *Trichogramma* females (<24 h). A previous study indicates that parasitism rates of *T. cacoeciae* females increased significantly on their second day of life [96]. Since the mean longevity of *Trichogramma* adults in tomato greenhouse is three days [28], the test was conducted for 48 h, resulting in a higher level of parasitism. This implies that our laboratory screening underestimated the efficiency of the moderately synovigenic species *T. cacoeciae*. The species is interesting for biological control because of various traits such as thelytoky (100% female offspring), high emergence, and ability to diapause [97]. Since *T. cacoeciae* is native to Germany, it could be released according to the Federal Nature Conservation Act [43].

In our study we identified three promising *Trichogramma* species as potential biocontrol agents against *T. absoluta*. However, this does not necessarily mean that other strains of these species achieve a similar efficiency: Various screenings showed that the level of parasitism can differ among strains of the same *Trichogramma* species [28,98,99,100]. We also recommend using a strain of European origin of *T. pintoi* for further field-testing in Europe.

For the next step, *T. nerudai*, *T. pintoi*, and *T. cacoeciae* should be tested under greenhouse conditions, also in comparison to *T. achaeae*, where possible. Promising strains may be less effective in greenhouses due to different environmental conditions [26,28,69]. However, a more recent study has shown that efficiency may be improved if rearing conditions (e.g., temperature) match the abiotic conditions of the target crop [101].

## 5. Conclusions

Based on our laboratory screening, we identified three *Trichogramma* strains as promising candidates for biological control of *T. absoluta* in Europe: *T. nerudai*, *T. pintoi*, and *T. cacoeciae* achieved a similar level of parasitism on potted tomato plants as our control species, *T. achaeae*. Further, our results indicated that the efficiency of moderately synovigenic species, such as *T. cacoeciae*, may be underestimated in various testing procedures. Thus, future screenings should consider the potential effects of variation in ovigeny more carefully.

## Figures and Tables

**Table 1:** Selected rearing strains of the genus *Trichogramma* and their origin.

Species	Rearing Strain ^1^	Origin	Year	Reference
*T. achaeae*	**ACA** BC14	Company 1 ^2^	unk. ^3^	unk.
*T. bourarachae*	**BOU** EG02	Egypt, olive	2002	[40]
*T. brassicae*	**BRA** DA	Company 2 ^2^	unk.	unk.
*T. cacoeciae*	**CAC** 1DE05	Germany	2005	BBA ^4^
*T. cordubensis*	**COR** PT93	Portugal, tomato, Noctuidae	1993	[34]
*T. dendrolimi*	**DEN** D90	Germany, apple, *Cydia pomonella*	1990	BBA
*T. evanescens*	**EVA** DE97K	Germany, cabbage, *Pieris* sp.	1997	BBA
*T. nerudai*	**NER** PT02	Portugal, olive	2002	[40]
*T. piceum*	**PIC** MD91	Moldova	1991	BBA
*T. pintoi*	**PIN** 3SY06	Syria	2006	BBA

^1^ Abbreviations of the rearing strains (printed in bold) are used in the following. Strains BOU and PIN are not of European origin, but these species occur in Europe. ^2^ Name of companies are known and can be communicated on request. ^3^ unknown. ^4^ Federal Biological Research Centre for Agriculture and Forestry (BBA), Darmstadt. Former strain collection of Dr. Sherif A. Hassan.

**Table 2:** Analysis of the ITS2 sequence of selected *Trichogramma* strains. Fragment sizes of PCR products were estimated based on the result of the gel electrophoresis.

Strain	Estimated Size PCR Product [bp]	Size Consensus Sequence [bp]	Species ^1^	Consensus [%]	Accession Number ^2^
ACA	630	317	*T. achaeae*	100.0	JF415936
BOU	650	* 78	*T. bourarachae*	96.2	DQ389072
BRA	520	431	*T. brassicae*	100.0	DQ314611
CAC	580	386	*T. cacoeciae*	99.5	EU547668
COR	530	349	*T.* sp.^3^	100.0	AY146636
DEN	520	423	*T. dendrolimi*	99.8	AF517576
EVA	550	395	*T. evanescens*	99.5	JF9204591
NER	750	346	*T. nerudai*	100.0	AY182756
PIC	850	441	*T. lingulatum* ^4^	99.8	AY244466
PIN	690	511	*T. pintoi*	99.8	JF920460

^1^ Species identification according to BLAST analysis. ^2^ Accession numbers of ITS2sequences published in GenBank giving the highest identity score in a BLAST alignment. ^3^ Only generic name available. ^4^ No reference sequence available for *T. piceum.* * Only a short part of the sequencing was used due to low quality.

**Table 3:** Active *Trichogramma* females (%) (= parasitizing at least one *T. absoluta* egg), emergence rate (%) of F1-progeny from parasitized host eggs and proportion of females (%) in the F1-progeny in the host acceptance test. Ten eggs of *T. absoluta* were offered to each *Trichogramma* female on a tomato leaf disc for 2 h and then incubated until emergence of the progeny. Arithmetic means ± standard errors (SE) are calculated from three trials per strain, each with 10 females, for active females (%) or from progeny per parasitizing female (up to 30 per strain) for emergence rate (%) and F1-females (%).

Strain	Active Females [%]	Emergence Rate [%]	Females F1 [%]
	(means ± SE)	(means ± SE)	(means ± SE)
ACA	63.3 ± 11.9	95.4 ± 2.6	63.6 ± 5.1
BOU	36.8 ± 2.7	100.0 ± 0.0	68.3 ± 4.2
BRA	46.7 ± 9.8	48.2 ± 13.0	66.8 ± 10.5
CAC	40.0 ± 8.2	100.0 ± 0.0	100.0 ± 0.0
COR	20.0 ± 8.2	66.7 ± 19.3	70.2 ± 5.2
DEN	13.3 ± 7.2	75.0 ± 21.7	38.9 ± 16.4
EVA	53.3 ± 9.8	100.0 ± 0.0	29.5 ± 8.4
NER	66.7 ± 7.2	98.1 ± 1.9	53.2 ± 7.3
PIC	20.0 ± 0.0	63.3 ± 18.5	77.2 ± 7.0
PIN	63.3 ± 13.6	88.8 ± 7.0	49.8 ± 6.2

**Table 4:** Active *Trichogramma* females (%) (=parasitizing at least one *T. absoluta* or one *S. cerealella* egg in 2 h) during the host preference test. Five eggs of the target host *T. absoluta* and five eggs of the rearing host *S. cerealella* were simultaneously offered to each *Trichogramma* female. Arithmetic means ± SE are calculated from three trials per strain, each with 10 females.

Strain	Active Females [%]	Active Females [%]
	Host: *T. absoluta*	Host: *S. cerealella*
	(means ± SE)	(means ± SE)
ACA	86.7 ± 7.2	86.7 ± 7.2
BOU	80.0 ± 0.0	73.3 ± 2.7
BRA	86.7 ± 2.7	80.0 ± 4.7
CAC	56.7 ± 2.7	73.3 ± 7.2
COR	66.7 ± 7.2	63.3 ± 14.4
DEN	76.7 ± 2.7	66.7 ± 11.9
EVA	70.0 ± 8.2	70.0 ± 8.2
NER	80.0 ± 0.0	76.7 ± 2.7
PIC	63.3 ± 5.4	80.0 ± 4.7
PIN	76.7 ± 11.9	80.0 ± 9.4

**Table 5:** Average egg lengths and widths of the target host *T. absoluta* and the rearing host *S. cerealella*. Arithmetic means ± SE are calculated from 20 measured eggs for each host species. Different letters within a column indicate significant differences among means (*t*-test, *p* < 0.05, *n* = 20).

Species	Egg Length [µm]	Egg Width [µm]
	(means ± SE)	(means ± SE)
*Tuta absoluta*	398.9 ± 3.8 b	260.6 ± 2.9 a
*Sitotroga cerealella*	622.1 ± 5.9 a	265.7 ± 2.9 a

## Supplementary Materials

The following material is available online at https://www.mdpi.com/2075-4450/11/6/357/s1: Microphotographs of male genital capsules of *Trichogramma* strains: Figure S1: Genital capsule of *T. brassicae* and *T. evanescens*, Figure S2: Genital capsule of strain COR, Figure S3: Genital capsule of *T. bourarachae* and *T. pintoi*, Figure S4: Genital capsule of strain PIC and *T. dendrolimi*, Figure S5: Genital capsule of *T. nerudai* and *T. achaeae*.
