# Supplementary material for: Suitability of European Trichogramma Species as Biocontrol Agents against the Tomato Leaf Miner Tuta absoluta
Source: Insects. 2020 Jun 8;11(6):357. doi: 10.3390/insects11060357 (PMC7349915; doi:10.3390/insects11060357)
Supplement: Supplementary file 1 [file insects-11-00357-s001.pdf]

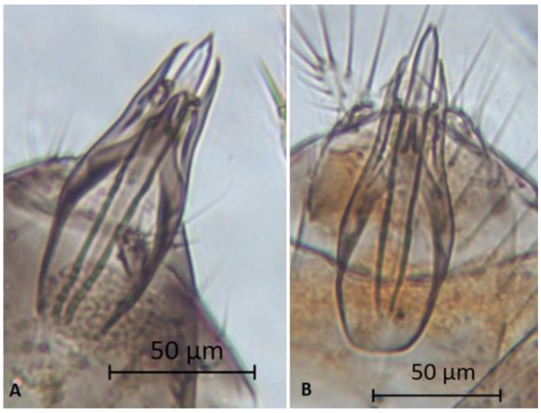

Figure S1: Genital capsule of *T. brassicae* (A) and *T. evanescens* (B), assigned to group *evanescens*.

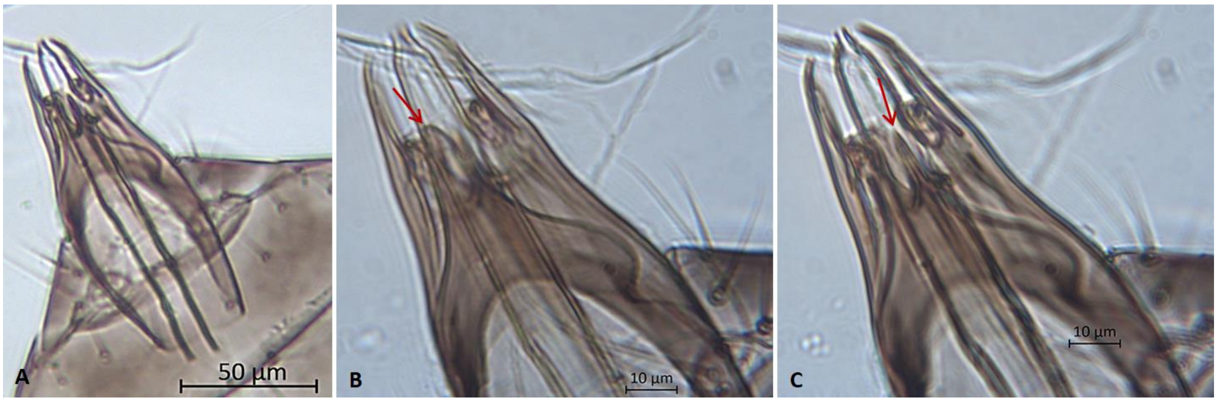

Figure S2: Genital capsule of strain COR, assigned to group *minutum*. Posterior extension of dorsal lamina (B) exceeds intervolsellar process (IVP) (C).

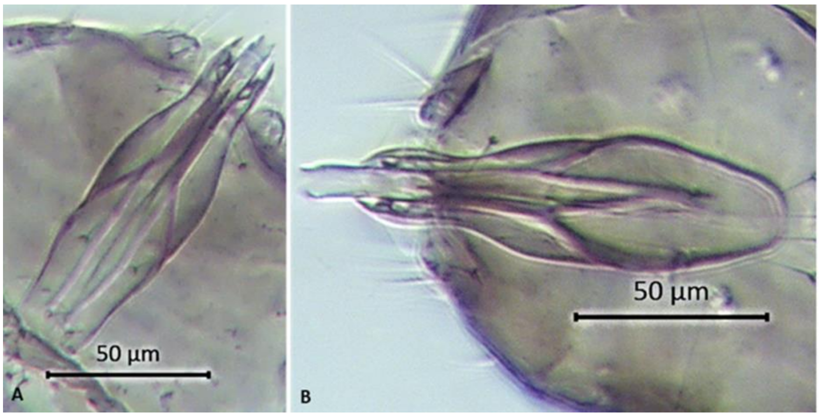

Figure S3: Genital capsule of *T. bourarachae* (A) and *T. pintoi* (B), assigned to group *perkinsi*.

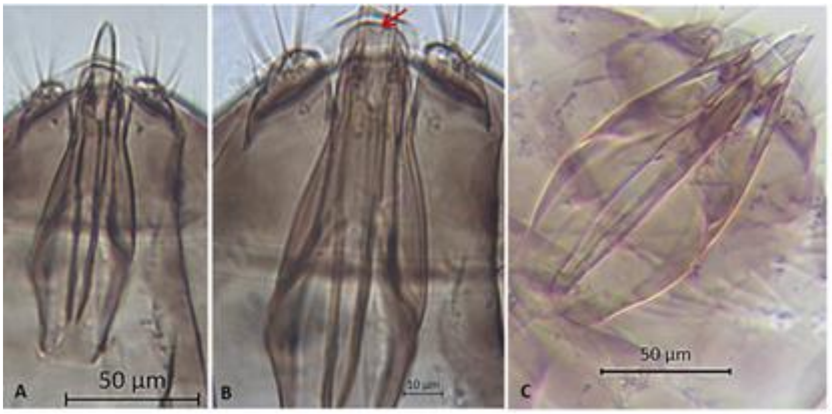

Figure S4: Genital capsule of strain PIC (A: Overall view, B: Posterior extention of dorsal lamina is broad and rounded and exceeds IVP, volsella and paramere), assigned to group *fasciatum*, and *T. dendrolimi* (C, group *dendrolimi*).

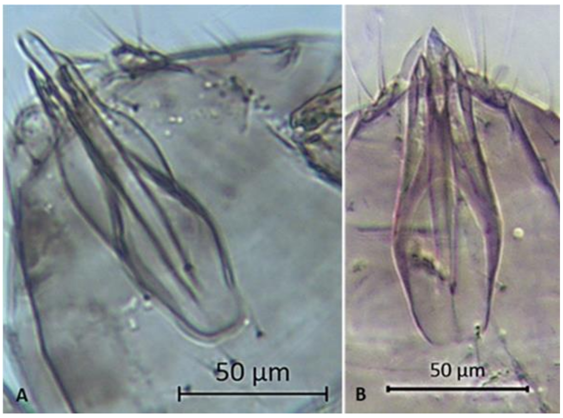

Figure S5. Genital capsule of *T. nerudai* (A) and *T. achaeae* (B).
